# Case report: One human *Streptococcus suis* occurred in Shandong Province, China

**DOI:** 10.1097/MD.0000000000032414

**Published:** 2022-12-23

**Authors:** Shuyu Chen, Renpeng Li, Xin Wang, Yuwei Liu, Zengqiang Kou, Qiang Wang

**Affiliations:** a College of Public Health, Weifang Medical University, Shandong, China; b Shandong Center for Disease Control and Prevention, China; c Department of Epidemiology, Weifang Medical University, Shandong, China.

**Keywords:** blood culture, case report, pork, *Streptococcus suis*

## Abstract

*Streptococcus suis* (*S suis*) is a major pathogen of bacterial infectious diseases, which can be transmitted to human beings through close contact with sick pigs or carriers, and can cause toxic shock, meningitis, septicemia, pneumonia, and other complications, with an extremely high mortality and disability rate. *S suis* is also an emerging zoonotic agent, mainly occurring in China, Thailand, and the Netherlands. This seriously threatens the health and family economy of patients.

**Case presentation:**

A 75-year-old man presented with a 1-day history of fever, vomiting, coughing, chills, and unconsciousness. He was admitted with the diagnosis sepsis and intracranial infection. At admission, hematologic studies showed a leukocyte count of 23.45 × 10^9^/L with 91% neutrophils. Chest computed tomography revealed double pneumonia. Blood cultures grew small colonies, which were identified as *S suis*. Antibiotic susceptibility testing revealed that the pathogen was susceptible to levofloxacin. And then, treatment with levofloxacin was implemented. Epidemiological investigations showed that the patient had eaten pork from a sick pig. When a patient with bacterial infection has a history of eating pork from sick pigs, human *S suis* infection should be taken seriously.

**Conclusion:**

Although human *S suis* infection generally presents as a sporadic disease, its high burden highlights the importance of epidemiological surveillance and health education regarding human *S suis* infection.

## 1. Introduction

*Streptococcus suis* (*S suis*) is an important pathogen of bacterial infectious diseases related to a variety of complications, including toxic shock, meningitis, septicemia, pneumonia, and so forth, and can be transmitted to human beings by direct contact with sick pigs or carriers.^[1]^ Human infection with *S suis* infection was first reported in 1954^[2,3]^{Field, 1954 #5;Wertheim, 2009 #7}. Human *S suis* cases were first identified in 1968 in Denmark.^[4]^ The disease was then diagnosed on a continuous basis in Europe, America, Asia, Africa, and Oceania.^[5,6]^ The fatality rate of human infection with *S suis* is approximately 12% worldwide.^[7]^ Meningitis is the most common human manifestation of *S suis* infection accounting for approximately 50% to 60% of reported cases,^[8]^ followed by sepsis, arthritis, endocarditis and endophthalmitis.^[9]^ The most frequently sequel is hearing loss, which occurred in 53% of cases.^[8]^ The expense of human infection with *S suis* may destroy the patients’ family economy.^[10]^ Owing to high pork consumption and small-scale swine feeding, human *S suis* infection is endemic in China.^[8]^ Although human *S suis* normally presents as a sporadic disease, there were 2 outbreaks in China.^[11,12]^ Toxic shock syndrome was a distinct and severe clinical presentation in these 2 outbreaks.^[9]^ The supposed reasons for outbreaks were slaughtering sick pigs and preparing carcasses of pigs that died of unknown causes.^[11]^ The fatality rate of human *S suis* infection in China is up to 18%.^[13]^ The features of human *S suis* and the state of pigs’ rearing and consumption in China highlight the need for accurate epidemiological surveillance of human *S suis.* In this article, we reported 1 case of human *S suis* infection in Shandong province, China.

## 2. Case presentation

The 75-year-old previously healthy man presented with a 1-day history of fever, vomiting, coughing, chills, and unconsciousness. Then, he was admitted by a local rural community hospital with a diagnosis of cerebral infarction on December 26, 2016. At admission to the local rural community hospital, the patient had a body temperature of 37.8°C and was treated with ozagrel. In order to achieve a definitive diagnosis and receive further treatment, the patient was transferred to the Weihai Central Hospital on December 27, 2016. He was admitted with a diagnosis of sepsis and an intracranial infection. At admission to Weihai Central Hospital, the patient had a body temperature of 36.4°C and a blood pressure of 126/83 mm Hg. Hematologic studies showed a leukocyte count of 23.45 × 10^9^/L with 91% neutrophils. Chest computed tomography revealed double pneumonia. Symptomatic treatment for anti-infection was implemented with levofloxacin, piperacillin sodium, and tazobactam sodium. Blood cultures grew gram positive cocci and antibiotic susceptibility testing revealed that the pathogen was susceptible to levofloxacin on December 29, 2016. Then, treatment with levofloxacin was initiated. On December 30, blood cultures grew small colonies that were identified as *S suis.* The patient made a recovery and was discharged voluntarily with hearing loss on January 16, 2017.

Epidemiological investigations showed that the patient had eaten pork from a sick pig before he fell ill. The sick pig was reared on a pig farm located outside the patient’s village.

This study was approved by the Ethics Review Committee of Weifang Medical University and the participants provided informed consent.

## 3. Discussion

In addition to rearing and slaughtering pigs, preparing and consuming pork are also risk factors for human *S suis* infection.^[5,14]^ In line with previous studies,^[9]^ the patient in this article had a history of eating pork from a sick pig. According to the history of eating pork and the result of blood cultures, it may be assumed that the patient was infected by *S suis* through eating the pork of a sick pig. Although it is forbidden to slaughter sick pigs and eat the pork of sick pigs in China,^[15]^ it still occurs to secretly slaughter sick pigs or privately eat the pork of sick pigs.^[16]^ There is a high likelihood that human infection with *S suis* will eat pork from sick pigs because both pork and contact with sick or dead pigs are risk factors of human *S suis* infection.^[3,5,11]^ Thus, it highlights the importance of epidemiological surveillance about human *S suis* in order to urgently treat human *S suis* cases. Health education about human *S suis* infection is also necessary for the sake of persuading people not to slaughter sick pigs or eat the pork of sick pigs. Then, human *S suis* infection may be prevented with the help of health education.

Consistent with previous studies,^[3,5,8,11]^ the patient in this article was diagnosed with sepsis. Sepsis and meningitis are common presentations of human *S suis* infection.^[5,8]^ Although the patient in this article was not specifically diagnosed with meningitis, it may be assumed that this patient also had meningitis according to the diagnosis of intracranial infection and the clinical presentation of fever, vomiting, coughing, chills, and unconsciousness. Meningitis and sepsis appeared in approximately 75% and 18% of reported cases, respectively.^[5]^ Approximately 60% of patients with meningitis have neurologic sequelae.^[6]^ In China, some patients had severe sepsis syndromes, and toxic shock syndromes, which were associated with high mortality.^[3,11]^ In addition to these short-term damages, human *S suis* may result in some long-term damages, including hearing loss with or without vestibular dysfunction.^[10]^ Approximately 53% of human *S suis* cases with meningitis may have hearing loss.^[17]^ Hearing function may be irreversible after 3 months.^[18]^ The patient in the current study also experienced hearing loss at admission, which is in accordance with previous reports.^[6,14,19]^ The fatality rate of human *S suis* cases was approximately 12% globally^[7,9]^ but up to 18% in some Chinese areas.^[13]^ Most patients infected by *S suis* are the major family income generators.^[10]^ If the health of these patients suffers substantial damage, their family income may significantly decrease. This is disastrous for their families. In addition to the damage to patient health, the direct economic expense per human *S suis* infection is about US$1635 which is greater than the patient’s annual per capita family income.^[10]^ The high burden of human *S suis* infection includes health damage, disabling sequelae, premature mortality, and economic expense. This high burden may destroy the patients’ families, which further highlights the need for epidemiological surveillance and health education about human *S suis* infection.

## 4. Conclusion

In this article, we reported 1 case of human *S suis*, which may result from eating the pork of a sick pig. Although human *S suis* infection generally presents as a sporadic disease, its high burden highlights the importance of epidemiological surveillance and health education regarding human *S suis* infection.

## Author contributions

Shuyu Chen, Renpeng Li, Zengqiang Kou and Qiang Wang were responsible for the analysis and interpretation of data, statistical analysis and writing of the article. Xin Wang and Yuwei Liu were responsible for the analysis and collection of data.

**Data curation:** Shuyu Chen, Renpeng Li, Xin Wang, Yuwei Liu.

**Investigation:** Shuyu Chen, Renpeng Li, Zengqiang Kou, Qiang Wang.

**Resources:** Zengqiang Kou.

**Software:** Xin Wang, Yuwei Liu, Zengqiang Kou.

**Writing – original draft:** Shuyu Chen, Qiang Wang.
